# Future anxiety and the motives for postponing parenthood: generational time perspective and life satisfaction as mediators

**DOI:** 10.3389/fpsyg.2024.1441927

**Published:** 2025-02-12

**Authors:** Małgorzata Szcześniak, Celina Timoszyk-Tomczak, Julia Łoś, Monika Grzeczka

**Affiliations:** Faculty of Social Sciences, Institute of Psychology, University of Szczecin, Szczecin, Poland

**Keywords:** future anxiety, postponed parenthood, delayed parenthood, generational time perspective, life satisfaction

## Abstract

**Introduction:**

Research to date has focused largely on the consequences of delayed parenting. However, little is known about the reasons and relevant mechanisms that are involved in people’s decisions to delay parenthood. The aim of our study was to verify how anxiety about the future relates to the motives for deferred parenthood and how this relationship is mediated by generational time perspective and life satisfaction.

**Methods:**

A total of 203 Polish adults between 18 and 40 years of age participated in the study. All of them were of reproductive age but did not yet have children. Respondents completed the following questionnaires: The Dark Future Scale, The Multidimensional Scale of Motives for Postponing Parenthood, The Generational Time Perspective Questionnaire, and The Riverside Life Satisfaction Scale.

**Results:**

The results obtained in the study show that future anxiety correlates significantly and positively with all six motives for postponing parenthood, postponed parenthood overall, and generational affectivity. It is also associated with the generational cognitive perspective at the level of tendency, and negatively with life satisfaction. According to our findings, in all seven models, at least one of the factors mediated the relationship between future anxiety and motives for postponing parenthood/its total score.

**Conclusion:**

The current study advances the understanding of how the subjective future time perspective is related to delayed parenthood through generational concern and reduced life satisfaction. Our findings may indicate that despite the importance of sociodemographic variables in postponing parenthood (e.g., lack of housing, financial instability, acquiring knowledge, education, etc.), variables related to personality and time perspective play a very important role in postponing the decision to have a child.

## 1 Introduction

Postponed parenthood consists of delaying the first childbearing toward later ages, into the mid or even late 30s (Sobotka, 2010; Waldenström, 2016). This growing phenomenon (Schlesinger and Schlesinger, 1989) from the early 1960s (Wilkie, 1981) is one of the most striking trends in contemporary reproductiveness (Skirbekk, 2022). According to different analyses, delaying parenthood characterizes high-income Western and East Asian countries (Sobotka, 2010; Thompson and Lee, 2011; Zacchini et al., 2022), middle-income developing societies of Latin America (Díaz, 2021; Díaz and Abufhele, 2023), and various groups of immigrants (Dupray and Pailhé, 2017).

Qualitative and quantitative findings indicate that many young people wait longer to have children because of multifaceted motives (Thompson and Lee, 2011; Zabak et al., 2023). Among numerous and nuanced factors accounting for delayed parenthood, scientists mention precarious social-economic conditions (e.g., lacking a partner, financial instability) (Martin, 2020), the need for maturity, personal growth, and autonomy (Boivin et al., 2009; Tydén et al., 2006), educational achievements, aspirations of self-realization (Díaz and Abufhele, 2023; Zabak et al., 2023), career advancement and women’s participation in the workplace (Zabak et al., 2023), technological advancements in assisted reproduction (e.g., egg freezing, donor insemination, *in vitro* fertilization) (Bellieni, 2012; NeJaime, 2017), a lack of fertility knowledge (Adachi et al., 2020; Okine et al., 2023), lifestyle goals (Datta et al., 2023), incompatibility of social roles (Datta et al., 2023; Nisén et al., 2022), and natural and anthropogenic causes of climate change (Schneider-Mayerson and Leog, 2020).

Besides the social and material preconditions of late parenthood, researchers also suggest the importance of individual and psychological determinants (Benzies et al., 2006; Bodin et al., 2021; Dion, 1995; Thompson and Lee, 2011). A factor that intuitively seems particularly salient in the context of parenting, although rarely discussed, is fear of the future, which can lead to indecision about starting a family. When people tend to anticipate negative outcomes and think about the future with worry (Carelli et al., 2011), they may consider undertaking more avoidant decision-making styles (Stolarski et al., 2018) and postpone being a parent. There are studies, especially those carried out among people with specific disabilities, that clearly document the link between anxiety and postponed parenthood. Such people often face fear for their own and their children’s future. For example, research conducted among visually impaired women revealed that they try to deal with their shortcomings to reduce their level of child-related anxiety (Commodari et al., 2022). In addition, potential parents with disabilities experience higher levels of anxiety while thinking of not being able to fulfill their parental responsibilities (Panuccio et al., 2020).

Less is known about relevant mechanisms that are involved in people’s decisions to delay parenthood. It seems that the generational time perspective, expressing interest in the life of future generations (Timoszyk-Tomczak and Próchniak, 2024), and life satisfaction, which is associated with a global cognitive assessment of one’s past life, might be such mechanisms. Therefore, the aim of this study was to explore the mediating role of generational time perspective and life satisfaction on the relationship between fear of the future and motives for postponing parenthood.

### 1.1 Future anxiety and postponed parenthood

Future time perspective, expressed through aspirations, expectations, fears, projects, and hopes, is considered an important psychological variable that facilitates imagining possible scenarios (Jannini et al., 2022), influencing people’s behavior (Coudin and Lima, 2011) and the choices they make (Tucholska et al., 2022). A review of the literature shows that reproductive decision-making, while it brings contentment and joy (Clarke et al., 2020), is often related to feelings of personal uncertainty (Reuter, 2019), conflicting emotions and attitudes about the transition to parenthood, and fear of taking an action that might be regretted (Lazarus et al., 2022).

Future anxiety is one of the basic elements of a negative future time perspective and refers to concern about unfavorable changes that may occur in the future (Zaleski, 1996). It has been found that future anxiety correlates positively with moving to independent adulthood (Koprowicz and Gumowska, 2022) and fear of childbirth (Zdybek and Haimann, 2017). This may be because anxious people make predictions of possible outcomes in a more negative than positive way (Sebaie et al., 2024).

Future anxiety, typically operationalized as a state of insecurity (Hwayan, 2020), a sense of uncertainty (Zaleski, 1996), and the fear of the unknown (Regnoli et al., 2024), reflects possible or anticipated adverse or dangerous events (Zaleski et al., 2019). People with anxious attitudes toward what is to come experience higher fear of forthcoming and distant threats, and lower levels of hope (Jannini et al., 2022). Likewise, the shift of childbearing to a later age is often accompanied by insecurities and uncertainties in life (Bodin et al., 2021). A lack of firm economic stability (Zabak et al., 2023), feeling of unpreparedness for parenthood (Avignon et al., 2023), and fear of childbirth (Avignon et al., 2023) contribute to postponing parenthood. It has been found that couples postponing their decision to have their first child are more concerned about the possible negative effects of parenthood on their freedom, career, and lifestyle than non-delayers (Dion, 1995). Climate change concerns are also reported by younger generations in the context of their reproductive plans and decisions (Schneider-Mayerson and Leog, 2020).

There is some empirical evidence that future anxiety and motives for deferred parenthood share common features. For example, Szcześniak et al. (2024) have noticed a positive association between anxiety and six motives for postponing the decision of parenthood (uncertainty about one’s skills in the context of parenthood, self-focus, perception of parenthood as a burden, fear of changes that the presence of the child may cause, financial concern, worries about the child’s future) and the overall score of postponing parenthood. This pattern of results was found both in Polish and American samples (Szcześniak et al., under review). Building upon the literature and previous empirical studies, we assumed that:

*H1*: Future anxiety is positively related to motives for postponing the start of childbearing and overall delayed parenthood.

### 1.2 Future anxiety and generational time perspective

The generational time perspective is understood as a cognitive-affective representation of the future relating to the life of a generation of people in the future, which the current generation of people will not live to see (Timoszyk-Tomczak and Próchniak, 2024). Previous research shows that fear of the future correlates with generational anxiety. Both constructs predict a negative future, but fear of the future is primarily cognitive in nature. It is associated with thoughts, fantasies, and images of what may happen, and only based on these images does anxiety appear (Zaleski, 1996). However, generational anxiety includes various difficult emotions regarding the future of subsequent generations in addition to anxiety, as well as anger and shame in connection with current events (Timoszyk-Tomczak and Próchniak, 2024).

Fear of the future is related to the personal future and to what extent personal plans can be implemented in a changing and hostile world (Zaleski, 1996). In contrast, generational anxiety goes beyond the personal future and reaches the future of the next generations. It may involve thinking in terms of the possibility of children and grandchildren, and people who will appear later, realizing their own plans. The future is the part of time that allows people to set long- and short-term goals and predict changes in their own capabilities, as well as their personal and broader social situations. It allows for locating goals in the near and distant future, which has motivational and behavioral consequences in the present (Lens et al., 2012).

The future can arouse hope and be a space for development (Zimbardo and Boyd, 2008), but it can also raise fears. Both aspects are not mutually exclusive (Zaleski, 1996). Recent years full of social and ecological disasters and experiences related to the pandemic may intensify these experiences (Pihkala, 2020). From a broader theoretical and empirical perspective, the predicted relationship between anxiety about future and generational anxiety can be based on the concept of micro and macro worries (Boehnke et al., 1998). The concept describes the structure of worries, indicating two aspects: the object of worry (the self or the in-group as examples of micro worries; the wider society and world as examples of macro worries) and the domain of worry, which relates to the area of life it concerns (e.g., environment, social relationships, achievement, economics). The authors of the concept emphasize that this structure refers to the cognitive dimension of worries (that includes an object and a domain of life) and to the affective dimension (which embraces differences in expectations related to the object and the domain of worries). In relation to the variables of interest to us, anxiety about the future refers to oneself as an object without reference to the area of life (Zaleski et al., 2019), while generational anxiety from the perspective of the self concerns specific areas of life, environmental changes, and social relations. Both constructs examine the same area, i.e., worries, of which anxiety is an important component. Given the existing evidence showing a link between future anxiety and generational affectivity, we hypothesized that:

*H2*: Future anxiety is positively related to generational time perspective in its two dimensions (affective and cognitive).

### 1.3 Future anxiety and life satisfaction

Life satisfaction is most commonly referred to as an individual evaluation of the quality of life based on one’s own perspective (Diener et al., 2013) and personal criteria (Chen et al., 2020). It is a stable indicator of subjective well-being (Shek and Li, 2016) and an important buffer against the negative effects of numerous stressors (Proctor et al., 2009). Theories of subjective well-being suggest that people derive life satisfaction from the objective conditions of their lives (e.g., contextual circumstances) and a variety of personality characteristics (Baird, 2010; Steckermeier, 2021). These theories may be complemented by the dual-pathway framework by Cunningham et al. (2015), according to which, time perspective (in our case, future anxiety) may directly affect life satisfaction.

There is an empirical consensus that anxiety, which is future-oriented (Eysenck et al., 2006), and life satisfaction are moderately negatively related (Headey et al., 1993). A growing body of research indicates that there is a consistent association between future anxiety and different aspects of well-being. For example, temporal negative affect (Chen et al., 2020; Mooney et al., 2017) is negatively correlated with life satisfaction. This means that people who view the future in an aversive way and have negative expectations (Rönnlund et al., 2021) tend to report lower levels of life satisfaction. Paolini et al. (2006) observe that unpleasant thoughts about the future significantly impede life satisfaction. Future-negative and future-confusional perspectives generate negative emotions and reduce psychological resources (Hao et al., 2024), leading to lower well-being.

Although the transition to parenthood is a normative change in people’s lives, it constitutes a source of stress and crises for couples, as well (Cowan and Cowan, 1995). Szcześniak et al. (under review) confirm that trait anxiety correlates moderately and negatively with resilience that, in turn, empowers childless individuals to grow in the face of adversity. Since resilience is considered a process of adaptation (Tamarit et al., 2023) and is positively associated with different domains of satisfaction, based on theoretical premises and previous findings, we considered that:

*H3*: Future anxiety is negatively associated with life satisfaction.

### 1.4 Generational time perspective and life satisfaction as mediators

In addition to the direct relationship, there is likely an indirect effect of future anxiety on delaying parenthood through generational time perspective and life satisfaction. Although there are no studies that clearly refer to the mediation process of both phenomena, except one study by Szcześniak et al. (2024), where the relationship between anxiety and postponed parenthood was mediated by maturity, there is some evidence that time perspective and life satisfaction may play such a role.

Starting with the generational time perspective, the basis for choosing this type of time as a mediator is that even if people generally do not focus much of their attention on the human beings who will come after them, they think about the future of their potential children and/or grandchildren, especially in the context of worrying about the world. Nowadays, there are more and more reasons for concern about climate change, and the concept of environmental anxiety or eco-anxiety appears in the literature. Research shows that anxiety about climate change can cause consequences at the psychological or spiritual level and lead to existential questions about existence and its meaning (Pihkala, 2020).

The rationale for selecting life satisfaction as a mediator is that this variable has been reported to have a mediating effect in the context of adverse life experiences. For example, Baruffol et al. (1995) noticed that life satisfaction explains why some people adapt to challenging or adverse situations without more frustration, whereas others do not. In another study (Luo et al., 2024), life satisfaction played a mediating role in the relationship between emerging adulthood characteristics and anxiety.

Taken together, the generational time perspective may coexist with lower subjective life satisfaction and, thus, increase doubts related to starting a family or planning children. This is because the anticipated changes do not provide a sense of stability, and the lack of stability in the context of various life challenges results in reduced subjective well-being (Szymańska, 2021). This, in turn, may lead to postponing the decision to have the first child. This is consistent with the perspective of Bradley and Corwyn (2004, p. 385), who imply that “life satisfaction reflects both the extent to which basic needs are met and the extent to which a variety of other goals are viewed as attainable.”

Moreover, a review of the literature on eco-anxiety shows that its severity may be associated with impaired functioning, symptoms of depression, anxiety, post-traumatic stress disorder, stress and insomnia, lower self-esteem of mental health, as well as reluctance to have children (Boluda-Verdú et al., 2022). All the above-mentioned constructs reflect reduced well-being, which is associated with lower levels of life satisfaction. Recent research also shows a sense of hopelessness and fear for the future of humanity among children and adolescents around the world (Hickman et al., 2021; Marks et al., 2021). Previous findings on the relationship between worries and mental health demonstrate that micro worries are strongly associated with poor mental health, whereas macro worries are unrelated to mental health or have little relationship with positive well-being (Boehnke et al., 1998). This suggests that the vision and expectations for the future influence responses about the impact of the climate on their current, hypothetical, and expected children. Planning to have children is also associated with increased anxiety about the future, although this relationship is complex and requires additional research (Schneider-Mayerson and Leog, 2020). Considering the previous studies, we proposed the following hypothesis:

*H4*: Generational time perspective and life satisfaction act as mediators in the relationship between future anxiety and dimensions of postponed parenthood/its overall score.

**Figure 1:**
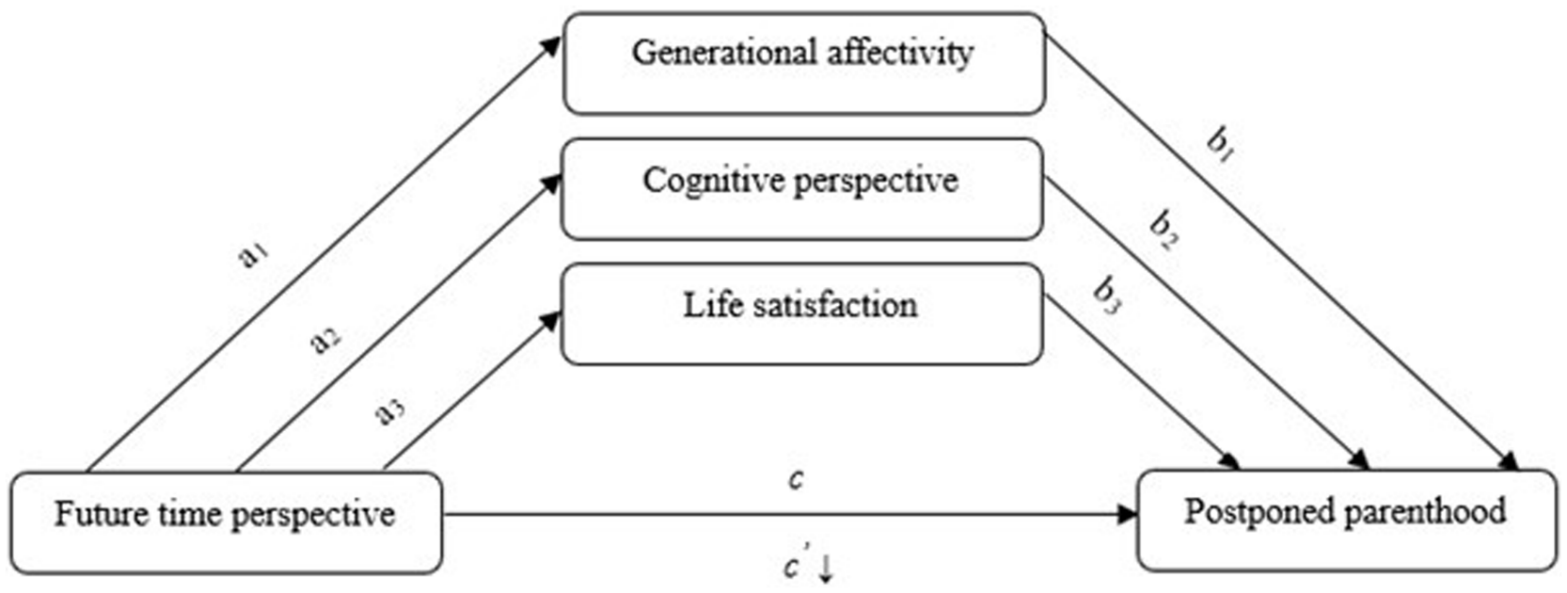
Hypothesized parallel model of the role of generational affectivity, cognitive perspective, and life satisfaction in the relationship between future time perspective and postponed parenthood.

The hypothesized parallel model is presented in Figure 1.

## 2 Research design, research tools, and statistical analyses

### 2.1 Participants and procedure

A total of 203 Polish adults participated in the study, 74.9% of whom were women (25.1% men). The age range of the sample was 18–40 years (*M* = 22.75; *SD* = 4.18). With respect to place of residence, 37.4% of the respondents were from cities between 150,000 and 500,000 inhabitants; 18.7% were from cities over 500,000 or from villages; 13.4% were from cities between 50,000 and 150,000 inhabitants; and 11.8% were from towns up to 50,000 inhabitants. Three-quarters of the participants (75.4%) stated that they chose to have a child before the age of 30. All subjects gave their written informed consent for taking part in the study.

The study was aimed at people who did not yet have children, but were of reproductive age, and was conducted via an online survey. The objectives of the study were communicated to the respondents along with instructions on its duration, anonymity, confidentiality, and the possibility of withdrawing at any time without any consequences. The study had project approval granted by the Research Ethics Committee of the Institute of Psychology at the University of Szczecin (No. 24/2023, 9th November 2023), and it was conducted following the guidelines provided in the Declaration of Helsinki.

### 2.2 Dark future scale

The Dark Future Scale (DFS) by Zaleski et al. (2019) is a short version of the Future Anxiety Scale. The DFS measures future anxiety, defined as a fear of anticipated adverse changes in the future (e.g., I am afraid that the problems troubling me now will continue for a long time). Participants evaluate their attitudes toward the future on a seven-point Likert scale, with 0 being *decidedly false* and 6 being *decidedly true*. In the current study, the scale presented very good internal reliability with Cronbach’s *α* = 0.897.

### 2.3 Multidimensional scale of motives for postponing parenthood

The Multidimensional Scale of Motives for Postponing Parenthood (MSMPP-18) by Szcześniak et al. (under review) is a measure to assess six motives to postpone the decision to have the first child: (1) feeling of uncertainty and incompetence involves a belief in one’s own unpreparedness to act as a parent (e.g., I am afraid I will not be able to cope with parental responsibilities); (2) self-focus refers to self-fulfillment (e.g., I am currently focusing on self-development); (3) parenthood as a burden implies fear of the sacrifices associated with having and caring for a child (e.g., Parenting is taxing); (4) fear of change reflects a fear of the unknown and potential negative consequences co-occurring with the birth of a child (e.g., A woman’s body changes unfavorably after pregnancy); (5) financial security concern is related to the perception of precarity and apprehension of not having sufficient resources to raise a child (e.g., The cost of raising a child is beyond my financial means); (6) worry about a child’s future stems from the fear that the child may suffer from climate change or wars (e.g., I do not want my child to live in unstable times). The MSMPP-18 can also be considered as one factor of deferred parenthood. The scale consists of 18 items, and each item is rated on a seven-point Likert scale (1 = *strongly disagree*, 7 = *strongly agree*). The values of internal consistency for all six dimensions and the total score were very good: (1) feeling of uncertainty and incompetence (α = 0.914); (2) self-focus (*α* = 0.929); (3) parenthood as a burden (*α* = 0.808); (4) fear of change (*α* = 0.786); (5) financial security concern (*α* = 0.914); (6) worry about a child’s future (*α* = 0.841); and the total score (*α* = 0.909).

### 2.4 Generational time perspective questionnaire

The Generational Time Perspective Questionnaire (GTPQ) by Timoszyk-Tomczak and Próchniak (2024) is a tool used to examine generational time perspective in two aspects: affective and cognitive. The affective dimension, with 5 items, diagnoses negative emotions (e.g., fear, anger, shame) toward threats that may occur in tens or hundreds of years (e.g., I am worried about what Earth will look like in the future). The cognitive dimension, which consists of 6 items, includes the concentration on the future manifested in an interest in the lives of people in future generations (e.g., I wonder how people will live in tens/hundreds of years from now). Respondents decide how much they agree with the statements using a 5-point Likert scale where 1 = *I strongly disagree* and 5 = *I strongly agree*. The tool is characterized by good reliability. In the present study, the internal consistency of the generational cognitive perspective is Cronbach’s α = 0.816, and the reliability of generational anxiety is α = 0.892.

### 2.5 Riverside life satisfaction scale

The Riverside Satisfaction with Life Scale (RSLS), developed by Margolis et al. (2019) and adapted into Polish by Adamczyk et al. (2020), is a brief, single dominant factor that assesses an individual’s satisfaction with their own life embraced as a whole. The questionnaire contains both regularly scored (e.g., I like how my life is going) and reverse-scored (e.g., I want to change the path my life is on) items. Respondents rate their agreement with each of the statements using a 7-point scale (1 = *strongly disagree* to 7 = *strongly agree*). In the current study, the RSLS showed good internal consistency with Cronbach’s α = 0.832.

### 2.6 Study size

To determine the optimum sample size in advance, an *a priori* power analysis was performed using G*Power 3.1.9.4 with a bivariate normal model correlation (Kang, 2021). A small effect size of 0.21 with an alpha of 0.05 and a power of 0.90 was set. The analysis showed that the total sample size would demand at least 187 participants. The rationale for using this value was based on meta-analyses that reveal that the average typical effect sizes in social psychology are equivalent to Pearson’s *r* = 0.21 (Richard et al., 2003).

### 2.7 Statistical analyses

The Shapiro–Wilk test was applied to evaluate the normality of the data. Since it showed that in all cases, the collected data was significantly different from the normal distribution, Spearman’s Rho correlations were conducted to assess the associations between future anxiety, motives for postponing parenthood and its overall score, generational affectivity, generational cognitive perspective, and satisfaction with life. A multivariable linear regression analysis was used to: (1) determine the degree of multicollinearity in a dataset; (2) test for unusual observations (outliers); (3) control for potential survey-related confounders and to examine whether they were affecting the direct association between the independent (future anxiety) and dependent (motives for postponed parenthood and its total score) variables.

A regression analysis was conducted to analyze the degree of near-linear dependence between the explanatory variables (Daoud, 2017). The tolerance statistics and the variance inflation factor (VIF) were used to calculate the variance inflation. The values showing a sign of multicollinearity were lower than 0.1 for tolerance and larger than 5 for VIF. Mahalanobis distance (*p* < 0.001) and Cook’s distance (higher than 1) were used to identify influential cases.

Sex, age, place of residence, a declaration about becoming a parent before the age of 30, and an indication of the most appropriate age to be a parent were included as potential confounding variables. With respect to sex, age, and place of residence, it has been found that there are some disparities between men/women, younger/older adults, and smaller/bigger cities concerning future anxiety and aspects of family functioning. For example, women scored higher than men on future anxiety (Awad et al., 2024; Bujnowska et al., 2019; Jannini et al., 2022) and on the current and ideal number of children (Thompson and Lee, 2011). Moreover, the level of future anxiety was significantly higher among younger emerging adults (18–20 years old) than their older counterparts (21–25 years old) (Koprowicz and Gumowska, 2022). The timing of the first birth was also related to the place of residence (Obeng Gyimah, 2003). Although the appropriate age to become parents should be autonomously chosen by a couple (Bellieni, 2012), according to Polish data released by Statista in 2023, the best age to have the first child for both women and men was between 25 and 29. All five prospective confounders were introduced in Step 1. The generational time perspective in two aspects (affective and cognitive) and life satisfaction were included in Step 2.

The data was analyzed using IBM SPSS Statistics (version 20). A multiple mediation was tested by the PROCESS macro for SPSS (Model No 4) with three parallel mediators, the Bootstrapping 5,000 technique, and 95% confidence intervals (CIs). Future anxiety was the independent variable, and the motives (feeling of uncertainty and incompetence—UNC, self-focus—SF, parenthood as a burden—BUR, fear of change—CH, financial security concern—FIN, worry about a child’s future—WOR) for postponing parenthood and its total score (MPP) were the dependent variables. Generational affectivity (GA), generational cognitive perspective (GCP), and life satisfaction (SAT) were included as mediators. Thus, seven pathways were proposed: FA → GA/GCP/SAT → UNC; FA → GA/GCP/SAT → SF; FA → GA/GCP/SAT → BUR; FA → GA/GCP/SAT → CH; FA → GA/GCP/SAT → FIN; FA → GA/GCP/SAT → WOR; and FA → GA/GCP/SAT → MPP. Mediation was considered successful if the 95% CIs did not encompass zero. Moreover, since it is recommended that not only the statistical significance of indirect effects but also the effect size of a given effect should be reported (Preacher and Kelley, 2011), we used the software application SmartPLS4 to calculate the magnitude of effect size (*v^2^*) for all mediation paths in seven models. Following the indications advocated by Ogbeibu et al. (2021) about the squared adjustment *v* effect, we assumed the value of 0.175 for a large effect, 0.075 for medium, and 0.01 for small as more suitable for indirect effects. All *v^2^* values lower than 0.01 were considered as an indication of no effect.

## 3 Results

### 3.1 Descriptive statistics

Descriptive statistics for the mean, standard deviation, values of skewness, kurtosis, the Shapiro–Wilk test of future anxiety, motives for postponing parenthood, generational anxiety, generational cognitive perspective, and satisfaction are presented in Table 1. The Shapiro–Wilk tests for normality showed that all factors presented a significant *p* value, except future anxiety.

**Table 1:** Descriptive statistics for the future anxiety, motives for postponing parenthood, generational anxiety, generational cognitive perspective, and satisfaction (*N* = 203).

	*M*	*SD*	Skewness	Kurtosis	Shapiro–Wilk	*p*
1. FA	18.38	8.13	−0.454	−0.789	0.948	0.237
2. UNC	12.23	5.94	−0.097	−1.276	0.929	0.001
3. SF	17.07	4.57	−1.188	0.652	0.825	0.001
4. BUR	16.88	4.09	−1.170	0.918	0.871	0.001
5. CH	11.88	4.88	−0.020	−0.996	0.968	0.001
6. FIN	16.98	5.00	−1.244	0.602	0.795	0.001
7. WOR	12.72	5.45	−0.190	−1.066	0.949	0.001
8. MPP	87.79	21.02	−0.454	−0.203	0.977	0.001
9. GA	18.56	4.98	−0.722	0.111	0.936	0.001
10. GCP	21.12	5.37	−0.584	0.229	0.965	0.001
11. SAT	27.16	7.16	−0.116	−0.450	0.986	0.036

FA, Future Anxiety; UNC, Uncertainty; SF, Self-Focus; BUR, Burden; CH, Change; FIN, Financial Concerns; WOR, Worry; MPP, Postponed Parenthood Total; GA, Generational Affectivity; GCP, Generational Cognitive Perspective; SAT, Satisfaction with Life.

### 3.2 Multicollinearity, outliers, and confounders

The VIF values ranged below 2 (between 1.047 and 1.644) and the lowest tolerance was 0.608, suggesting no presence of multicollinearity in the data. The Mahalanobis distance for multivariate outlier detection revealed a chi-squared value of less than 0.001 in only two of the 203 cases. Likewise, Cook’s distance values (between 0.000 and 0.279) indicated that the outliers were not problematic.

The linear regression model showed that five categories included in Step 1 explained 24.7% of the variance (R^2^ = 0.247): sex (*β* = −0.162, t = −2.744, *p* = 0.007), age (*β* = −0.138, *t* = −2.420, *p* = 0.016), place of residence (*β* = −0.009, t = −0.159, *p* = 0.874), declaration about having a child before the age of 30 (*β* = 0.320, *t* = 5.643, *p* = 0.001), and indication of the most appropriate age to decide to have the first child (*β* = 0.088, *t* = 1.579, *p* = 0.116). Three of the above-mentioned variables had a significant effect, suggesting that motives for postponed parenthood may decrease among men and older adults, and increase among those people who consider having a child before the age of 30. Future perspective (*β* = 0.305, *t* = 4.404, *p* = 0.001), generational affectivity (*β* = 0.207, t = 3.153, *p* = 0.002), generational cognitive perspective (*β* = 0.081, *t* = 1.346, *p* = 0.180), and satisfaction with life (*β* = −0.041, *t* = −0.641, *p* = 0.523) predicted a significant amount of the variance (43.8%; *F*(202,9) = 16.698, *p* = 0.001), even after controlling for the effects of confounders.

### 3.3 Correlations

Table 2 illustrates the bootstrap correlation coefficient 95% confidence intervals between variables considered in the study: future anxiety, motives for postponed parenthood and its total score, generational affectivity, generational cognitive perspective, and satisfaction with life. The results are mostly consistent with the hypotheses. Future anxiety correlates significantly and positively with five motives for postponing parenthood (except self-focus) and postponed parenthood overall (H1). It also correlates significantly and positively with generational affectivity and with the generational cognitive perspective at the level of tendency (H2). Future anxiety is associated negatively with life satisfaction (H3).

**Table 2:** Correlations of the study variables (*N* = 203).

	FA	UNC	SF	BUR	CH	FIN	WOR	MPP	GA	GCP	SAT
1. FA	1										
2. UNC	0.42*** [0.295;0.542]	1									
3. SF	0.15* [0.000;0.294]	0.17* [0.034;0.294]	1								
4. BUR	0.37*** [0.241;0.498]	0.49*** [0.383;0.584]	0.44*** [0.438;0.662]	1							
5. CH	0.35*** [0.210;0.477]	0.58*** [0.483;0.672]	0.26*** [0.128;0.383]	0.51*** [0.404;0.605]	1						
6. FIN	0.40*** [0.273;0.521]	0.41*** [0.293;0.520]	0.470*** [0.323;0.596]	0.44*** [0.302;0.563]	0.28*** [0.161;0.404]	1					
7. WOR	0.26*** [0.124;0.396]	0.41*** [0.287;0.526]	0.15* [0.005;0.283]	0.39** [0.257;0.504]	0.48*** [0.363;0.595]	0.29*** [0.145;0.409]	1				
8. MPP	0.47*** [0.341;0.589]	0.76*** [0.698;0.805]	0.59*** [0.477;0.674]	0.78*** [0.710;0.831]	0.75*** [0.678;0.803]	0.68*** [0.593;0.755]	0.66*** [0.580;0.737]	1			
9. GA	0.31*** [0.162;0.442]	0.23** [0.093;0.359]	0.27*** [0.108;0.425]	0.36*** [0.206;0.493]	0.24** [0.106;0.363]	0.33*** [0.171;0.472]	0.42*** [0.297;0.537]	0.44*** [0.307;0.551]	1		
10. GCP	.133^t^ [−0.021;0.280]	−0.038 [−0.184;0.108]	0.23** [0.062;0.381]	0.15* [0.007;0.305]	0.088 [−0.052;0.225]	0.18** [0.033;0.326]	0.21** [0.055;0.349]	0.19** [0.028;0.334]	0.38*** [0.215;0.527]	1	
11. SAT	−0.47*** [−0.584;-0.347]	−0.29*** [−0.420;−0.159]	0.14* [0.001;0.271]	0.02 [−0.118;0.146]	−0.25*** [−0.385;−0.114]	-0.11 [−0.240;0.037]	−0.16* [−0.301;−0.002]	−0.17* [−0.307;−0.038]	0.08 [−0.070;0.220]	0.08 [−0.072;0.238]	1

*** *p* < 0.001; ** *p* < 0.01; * *p* < 0.05; ^t^ 0.05 < *p* < 0.1; FA, Future Anxiety; UNC, Uncertainty; SF, Self-Focus; BUR, Burden; CH, Change; FIN, Financial Concerns; WOR, Worry; MPP, Postponed Parenthood Total; GA, Generational Affectivity; GCP, Generational Cognitive Perspective; SAT, Satisfaction with Life.

### 3.4 Mediating effect of generational time perspective and life satisfaction

Table 3 presents the results of the PROCESS macro for SPSS of the relationship between future anxiety and the motives for postponing parenthood. Bootstrap estimates for the indirect effect, based on 5,000 bootstrap samples, showed that zero was outside of the lower and upper bounds of the CIs in all seven models. More precisely, the study found that two factors (generational affectivity and life satisfaction) mediated the relationship between the predictor (future anxiety) and the outcome variables (motives for postponing parenthood/its total score) in five models: FA → GA/SAT → UNC; FA → GA/SAT → BUR; FA → GA/SAT → CH; FA → GA/SAT → FIN; FA → GA/SAT → WOR. Moreover, life satisfaction alone was a mediator in the relationship between future anxiety and self-focus—FA → SAT → SF. Generational affectivity alone was a mediator in the relationship between future anxiety and the total score of postponed parenthood—FA → GA → MPP. The generational cognitive perspective was not a mediator in any model.

**Table 3:** Role of the generational affectivity, generational cognitive perspective, and life satisfaction in the relationship between future time perspective and dimensions of postponed parenthood/overall score (*N* = 203).

	a_1_, a_2_, a_3_ paths	b_1_, b_2_, b_3_ paths	c path	c’ path	Indirect effect	B(SE)	Lower CI	Upper CI
FA → GA/GCP/SAT → UNC	0.19***	0.24**	0.30***	0.22***	0.0453	0.0201	0.0126	0.0924
	0.09 (in)	−0.16*			−0.0139	0.0112	−0.0404	0.0022
*R^2^* = 0.23, *F*(4, 198) = 14.46, *p* = 0.001	−0.44***	−0.11*			0.0524	0.0269	0.0032	0.1097
FA → GA/GCP/SAT → SF	0.19***	0.13(in)	0.08*	0.10*	0.0260	0.0159	−0.0012	0.0616
	0.09 (in)	0.11 (in)			0.0098	0.0093	−0.0031	0.0328
*R^2^* = 0.13, *F*(4, 198) = 7.22, *p* = 0.001	−0.44***	0.12*			−0.0540	0.0177	−0.8990	−0.0203
FA → GA/GCP/SAT → BUR	0.19***	0.18**	0.18***	0.20***	0.0341	0.0149	0.0096	0.0681
	0.09 (in)	0.00 (in)			0	0.0059	−0.0127	0.0124
*R^2^* = 0.23, *F*(4, 198) = 14.81, *p* = 0.001	−0.44***	0.10*			−0.0445	0.0150	−0.0740	−0.0151
FA → GA/GCP/SAT → CH	0.19***	0.18*	0.21***	0.13**	0.0332	0.0169	0.0054	0.0715
	0.09 (in)	0.00 (in)			0.0003	0.0064	−0.0119	0.0152
*R^2^* = 0.16, *F*(4, 198) = 9.30, *p* = 0.001	−0.44***	−0.11*			0.0466	0.0220	0.0053	0.0917
FA → GA/GCP/SAT → FIN	0.19***	0.19**	0.25***	0.21***	0.0361	0.0198	0.0028	0.0803
	0.09 (in)	0.05 (in)			0.0048	0.0070	−0.0069	0.0219
*R^2^* = 0.21, *F*(4, 198) = 13.20, *p* = 0.001	−0.44***	0.03 (in)			−0.0123	0.0213	−0.0555	−0.0282
FA → GA/GCP/SAT → WOR	0.19***	0.43***	0.18***	0.04 (in)	0.0817	0.0366	0.0701	0.2130
	0.09 (in)	0.06 (in)			0.0053	0.0082	−0.0081	0.0248
*R^2^* = 0.22, *F*(4, 198) = 14.02, *p* = 0.001	−0.44***	−0.12*			0.0526	0.0270	0.0045	0.1093
FA → GA/GCP/SAT → MPP	0.19***	1.37***	1.21***	0.91***	0.2563	0.0839	0.0792	0.4435
	0.09 (in)	0.07 (in)			0.0063	0.0256	−0.0438	0.0665
*R^2^* = 0.32, *F*(4, 198) = 22.79, *p* = 0.001	−0.44***	−0.09 (in)			0.0407	0.0801	−0.1100	0.2049

*** *p* < 0.001; ** *p* < 0.01; * *p* < 0.05; (in), insignificant; FTP, Future Anxiety; UNC, Uncertainty; SF, Self-Focus; BUR, Burden; CH, Change; FIN, Financial Concerns; WOR, Worry; MPP, Postponed Parenthood Total; GA, Generational Affectivity; GCP, Generational Cognitive Perspective; SAT, Satisfaction with Life.

Although mediation was supported by the CIs in a significant proportion of the models, the lower bounds of the reported intervals were close to zero in six of eleven cases, thus suggesting that the effect sizes in these models could be weak. In fact, as presented in Table 3, only one of eleven significant mediations (as indicated by lower and upper CIs) showed a small effect size of 0.015 (FA → GA → WOR), slightly overpassing the value of 0.01 indicated by Ogbeibu et al. (2021) as a threshold for a small effect. The remaining significant models displayed negligible effects, providing information that the magnitude of the effect sizes was low. It is important to highlight that all values of *v*^2^ were insignificant (except one with a small effect mentioned before) despite values *p* and CIs being significant. From a theoretical perspective, these findings can be potentially very meaningful (Agler and De Boeck, 2017) and indicate that a tendency to anxiously perceive the future may lead to higher generational affectivity and lower life satisfaction, which in turn may result in various motives for postponing parenthood (H4). At the same time, from a practical perspective, the resulting effect sizes should be interpreted with caution as they are very small.

## 4 Discussion

The study aimed to analyze the relationship between future anxiety and motives for postponing parenthood, considering the mediating role of generational time perspective and/or life satisfaction. We hypothesized that future anxiety is positively related to motives for postponing parenting decisions (H1), future anxiety is positively related to generational time perspective in its two dimensions (affective and cognitive) (H2), future anxiety correlates negatively with life satisfaction (H3), and that generational time perspective and life satisfaction mediate the direct relationship between future anxiety and motives for postponing parenthood (H4).

With respect to hypothesis H1, which was largely confirmed (except self-focus), our findings suggest that personal preoccupation with unfavorable changes in the future coexists with feelings of one’s own unpreparedness to act as a parent, fear of the sacrifices associated with being a parent, fear of change, perception of financial precarity, and worry about the child’s future. Because of the lack of empirical evidence on future anxiety and motives for postponing parenthood, we discuss our findings in the light of research on related concepts and phenomena. A range of studies supports that future negative and future confusion correlate negatively with confidence in one’s own worth (Zhi et al., 2021). Other studies show that the intense experience of anxiety contributes to a threat to one’s self-image and is directly related to uncertainty about one’s self (Joiner et al., 1999; Sowislo and Orth, 2013). The relationship between future anxiety and self-focus can be justified by the positive correlation between negative affect and focus on numerous aspects of the self. The positive correlation of future anxiety with burden and fear of change can be confirmed by the literature on the nature of anxiety related to parenthood. Given that being a parent is considered the most difficult job (Nomaguchi and Milkie, 2020; Panayiotou and Vrana, 2004) anxiety about parenting may lead to its postponement. In fact, previous analyses show that a reluctance to sacrifice and the perception of a significant burden associated with motherhood are two of the main motives of voluntarily childless women (Wilak, 2023). Some young people have a “belief that children detract from the marital relationship by interfering in the leisure time and intimacy of couples” (Hird and Abshoff, 2000, p. 352). If future anxiety means a fear of unfavorable changes, such a concern may result in the expectation that something bad may occur (Zaleski, 1996), and consequently, generate the perception of parenthood as a burden and unwanted change. When it comes to the relationship between anxiety and economic concern, this knowledge is well established, especially in the context of COVID-19 research. It has been proved by different scientists that higher levels of anxiety are significantly associated with financial concern (Haliwa et al., 2021; Oh et al., 2021). Recent findings provide evidence that individuals with high neuroticism scores tend to report higher financial distress (Fachrudin and Latifah, 2022), and emotional instability is one of its strongest predictors (Xu et al., 2015). Finally, some studies confirm the link between anxiety and worry about a child’s future. According to various researchers (Galway and Field, 2023), young people who have negative thoughts and experience negative emotions in connection with climate change (e.g., fear, sadness, anxiety, helplessness, etc.) express doubts about having children. This may be due to the relationship found in previous studies, which indicated that perceived threat correlates with higher levels of worry (Berenbaum et al., 2007).

Regarding hypothesis H2, which was fully confirmed, future anxiety significantly and positively correlates with affective generational time perspective and with the cognitive dimension. This means that young people with increased anxiety about the future anticipate negative consequences regarding their living conditions and the possibility of implementing their plans, not only for their own, but also for future generations. The results obtained in our study are consistent with previous studies, in which the affective dimension of generational time perspective correlated more strongly with future anxiety than the cognitive dimension (Timoszyk-Tomczak and Próchniak, 2024). The positive relationship between these variables is not surprising because all three relate to time perspective, involving anxiety about future events. However, both the theoretical foundations and the low strength of the correlation between them indicate that they are not identical constructs. While future anxiety refers to thoughts about the distant future concerning the person who imagines it (Zaleski, 1996), generational time perspective refers to potentially difficult events that may impact future generations (Timoszyk-Tomczak and Próchniak, 2024).

The third hypothesis, regarding a negative relationship between future anxiety and life satisfaction (H3), was fully confirmed and is, therefore, consistent with the results obtained in previous studies. Fear of the future indicates a predominance of negative attitudes toward the future over positive ones and refers to the anticipation of dangerous and unfavorable changes (Zaleski, 1996). Negative attitudes toward the future are associated with lower well-being and lower life satisfaction, which is confirmed by meta-analyses (Kooij et al., 2018).

The fourth hypothesis, about the mediating role of generational time perspective and life satisfaction in the relationship between future anxiety and postponed parenthood (H4), was largely confirmed. The findings obtained in the current research show that future anxiety is associated with postponing parenthood through fear of how future generations will function and life satisfaction. Although there are no analyses relating to possible conditions for postponing parenthood related to civilizational changes, future time perspective, and anxiety about upcoming changes, research in eco-anxiety indicates that its severity may be associated, among other things, with a deterioration of mental health and reluctance to have children (Boluda-Verdú et al., 2022). If the fear for future generations increases and is related to expected problematic living conditions or difficulties in achieving goals, it may limit the creation of plans for the future and decrease life satisfaction, especially if these plans are related to having children. The obtained results indicate that people who are anxious about the future, with higher generational anxiety and lower levels of life satisfaction, tend to postpone parenthood. It is well documented that the skills and opportunities to plan for future desired outcomes are critical to well-being, motivation, and behavior (Kooij et al., 2018). Future time perspective is associated with affective traits and personality predispositions. People who are open, extroverted, conscientious, full of hope, and positive have a longer future perspective and, therefore, are more motivated to implement different activities (Kooij et al., 2018).

It needs to be noted that our mediational results, although demonstrating the existence of mediation supported by CIs around the indirect effect, should be treated with caution due to the small magnitude of effect sizes shown in the study. It can be assumed that the mediators we chose as variables that could explain the relationship between future anxiety and postponed parenthood, although important and theoretically justified, are not necessarily essential ones. In fact, as Funder and Ozer (2019) observe, human psychology is naturally complex, and not all constructs have a large effect on mental actions, emotions, or behaviors. Moreover, it is empirically confirmed (Walters, 2019) that effect sizes in the context of mediation analysis are almost always small. Since there are no studies on a similar topic in which authors reported effect sizes for the indirect effects, it is difficult to draw far-reaching conclusions.

### 4.1 Limitations

Undoubtedly, our study adds new knowledge about the direct relationship between future anxiety and motives for postponing parenthood, and indicates the mediating role of generational affectivity and life satisfaction in this relationship. However, it also presents some limitations. Due to the cross-sectional nature of the study, we cannot talk about the causal type of direct relationships and indirect effects through the demonstrated mediations. Although the rationale for the direction of these relationships was based on previous, particularly predictive, research, longitudinal studies are needed to confirm this trajectory. The findings can also be affected by the confounding variables that we included in the study. Motives for postponed parenthood may be influenced not only by future anxiety, generational affectivity, and life satisfaction, but also by sex, age, and the consideration of having a child before or after the age of 30. The results regarding the magnitude of effect size seem to support this perspective.

## 5 Conclusions and implications

While research shows that anxiety is a predictor of indecision or even inhibits decision-making (Jia et al., 2020), we know less about the phenomena that may play an important role in the relationship between future anxiety and motives for postponing parenthood (Szcześniak et al., 2024). For this reason, the current study advances the understanding of how the subjective future time perspective is related to delayed parenthood through generational concern and reduced life satisfaction. It also has important implications for the developmental aspects of emerging adulthood, which is considered the most unstable period of the life span (Arnett et al., 2014). Our findings may indicate that despite the importance of sociodemographic variables in postponing parenthood (e.g., lack of housing, financial instability, acquiring knowledge, education, etc.), variables related to personality and time perspective may also play a very important role in postponing the decision to have a child.

Moreover, the future can be a source of worries about very personal and global situations (Zaleski, 1996). Further research requires clarification of what type of anxiety about the future is more important for postponing parenthood. Further analyses could be aimed at understanding the structure of anxiety about the future, and what type of anticipated threats are the most inhibiting in making decisions about having children. Are these concerns about one’s own skills related to the role of a parent, social and living restrictions, fears of losing previous opportunities and a sense of freedom, or potential threats to future generations and the world? Research could also include identifying factors that minimize anxiety about the future. This may be important for individual development and the realization of life goals characteristic of emerging adulthood, and it could also help regulate fertility, which decreases precisely where conditions and opportunities for children are greater, i.e., in high-income countries and developing societies.

## Data Availability

Publicly available datasets were analyzed in this study. This data can be found at: https://osf.io/gpb63/?view_only=d1623ffca4264589bb80cd8b0e0db200.
